# Hyaluronic Acid-Binding, Anionic, Nanoparticles Inhibit ECM Degradation and Restore Compressive Stiffness in Aggrecan-Depleted Articular Cartilage Explants

**DOI:** 10.3390/pharmaceutics13091503

**Published:** 2021-09-18

**Authors:** Marcus Deloney, Parssa Garoosi, Vanessa F. C. Dartora, Blaine A. Christiansen, Alyssa Panitch

**Affiliations:** 1 Biomedical Engineering Department, 451 E. Health Sciences Dr. Room 2303, University of California Davis, Davis, CA 95616, USA; madeloney@ucdavis.edu (M.D.); pdgaroosi@ucdavis.edu (P.G.); vfcdartora@ucdavis.edu (V.F.C.D.); 2 Department of Surgery, School of Medicine, University of California Davis, Sacramento, CA 95817, USA; 3 Lawrence J. Ellison Musculoskeletal Research Center, Department of Orthopedic Surgery, University of California Davis Health, 4635 2nd Avenue, Suite 2000, Sacramento, CA 95817, USA; bchristiansen@ucdavis.edu

**Keywords:** *N*-isopropylacrylamide, core-shell nanoparticle, thermosensitive, targeted drug delivery, biotherapeutic

## Abstract

Joint trauma results in the production of inflammatory cytokines that stimulate the secretion of catabolic enzymes, which degrade articular cartilage. Molecular fragments of the degraded articular cartilage further stimulate inflammatory cytokine production, with this process eventually resulting in post-traumatic osteoarthritis (PTOA). The loss of matrix component aggrecan occurs early in the progression of PTOA and results in the loss of compressive stiffness in articular cartilage. Aggrecan is highly sulfated, associates with hyaluronic acid (HA), and supports the compressive stiffness in cartilage. Presented here, we conjugated the HA-binding peptide GAHWQFNALTVRGSG (GAH) to anionic nanoparticles (hNPs). Nanoparticles conjugated with roughly 19 GAH peptides, termed 19 GAH-hNP, bound to HA in solution and increased the dynamic viscosity by 94.1% compared to an HA solution treated with unconjugated hNPs. Moreover, treating aggrecan-depleted (AD) cartilage explants with 0.10 mg of 19 GAH-hNP restored the cartilage compressive stiffness to healthy levels six days after a single nanoparticle treatment. Treatment of AD cartilage with 0.10 mg of 19 GAH-hNP inhibited the degradation of articular cartilage. Treated AD cartilage had 409% more collagen type II and 598% more GAG content than untreated-AD explants. The 19 GAH-hNP therapeutic slowed ECM degradation in AD cartilage explants, restored the compressive stiffness of damaged cartilage, and showed promise as a localized treatment for PTOA.

## 1. Introduction

Post traumatic osteoarthritis (PTOA) accounts for 12.5% of the over 32.5 million cases of osteoarthritis (OA) within the United States [1]. PTOA is characterized by inflammation of the joint and degradation of articular cartilage [2]. The extracellular matrix (ECM) of cartilage is primarily composed of proteoglycans (4–7% wet weight) and collagen type II (15–22% wet weight), and their interactions significantly control the biology of cartilage [3]. The most abundant proteoglycan in articular cartilage is aggrecan, which is composed of a core protein with covalently bonded sulfated glycosaminoglycan (GAG) chains. The sulfated GAG chains within aggrecan provide a high density of anionic charge, generating an osmotic gradient and enabling cartilage to retain water. This gives articular cartilage its compressive stiffness [4]. Aggrecan, the most prevalent proteoglycan in cartilage, is anchored to hyaluronic acid (HA) within the ECM of articular cartilage [3,4,5]. Aggrecan protects the cartilage ECM by interfering with the ability of collagenases to permeate the cartilage and cleave collagen type II [5]. However, following joint trauma, early loss of aggrecan as a result of digestion by upregulated aggrecanase causes the anionic GAGs to diffuse from the cartilage [5,6]. The loss of the GAGs leads to a reduced osmotic gradient and associated compressive stiffness within cartilage [5,7]. Binding anionic polymers to aggrecan-depleted HA may therefore have the potential to restore the mechanical function of articular cartilage and protect against further damage.

Currently, there is no FDA-approved disease-modifying therapeutic to treat OA. The present gold standards to treat OA and PTOA focus on alleviating the pain associated with OA and include intra-articular injections of non-steroidal anti-inflammatory drugs (NSAIDs), corticosteroids, glucosteroids, and viscosupplements. However, NSAIDs, corticosteroids and glucosteroids are non-specific and only address joint inflammation and pain, not the damaged cartilage itself [8,9]. Viscosupplements, commonly used once the patient complains of pain, aim to increase mobility and reduce discomfort. However, viscosupplements only delay surgical intervention and have conflicting evidence of efficacy [10,11]. The discovery and subsequent use of peptides able to bind to components within the ECM of articular cartilage present a solution to treat early-stage joint trauma and potentially prevent the progression of PTOA. Peptide-conjugated therapeutics are able to bind to aggrecan-depleted HA and can restore the compressive stiffness of osteoarthritic cartilage [7,12,13]. Previous studies utilized the HA-binding peptide GAH conjugated to polymers to inhibit the progression of OA. One study conjugated GAH and a collagen type II binding peptide to poly(ethylene glycol) (PEG) and slowed the degradation of articular cartilage following anterior cruciate ligament transection [14]. Other studies conjugated GAH to the sulfated GAG chondroitin sulfate (CS) to mimic aggrecan function. The GAH-CS conjugate bound to HA and restored the compressive stiffness of AD cartilage [7,12,13]. Further, CS-GAH slowed the release of GAGs into the media in ex vivo cartilage explants and suppressed matrix metalloprotease activity [7,15]. We aimed to build upon previous GAH-CS conjugates by using an anionic, sulfated, polymeric nanoparticle conjugated with GAH to inhibit the progression of OA.

**Figure 1:**
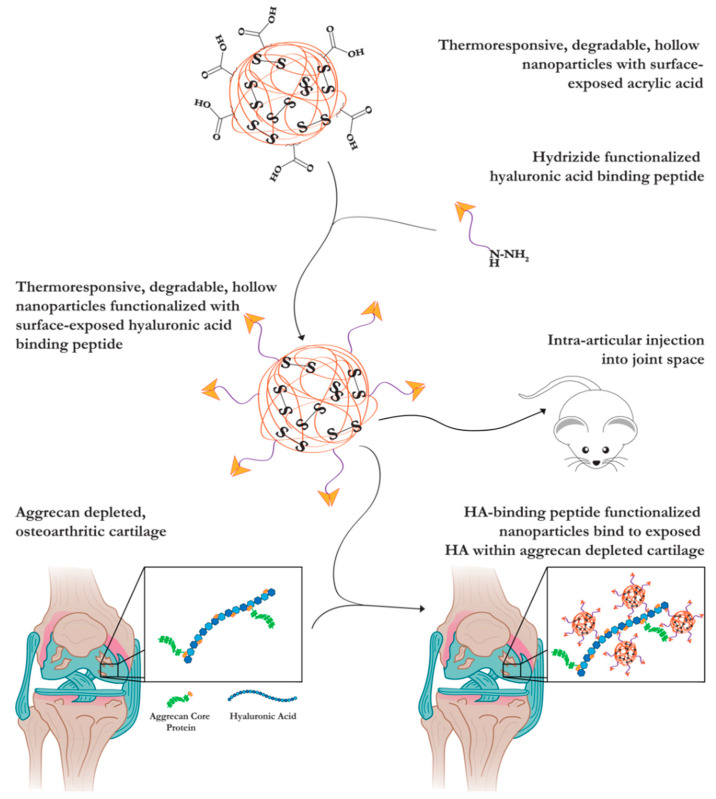
Schematic of the described studies. AAc polymerized into the nanoparticle shell served as the anchoring point of hyaluronic acid-binding peptide (GAH) conjugation, termed GAH-hNP. The GAH-hNP therapeutic treated aggrecan-depleted (AD) cartilage explants and was retained within the joint space of rats. The image was created using BioRender (access date: 25 January 2021).

In this study, we modified the recently developed anionic, polymeric, hollow nanoparticle (hNP) composed of *N*-isopropyl acrylamide (NIPAm), 2-acrylamido-2-methyl-1-propanesulfonic acid (AMPS), *N*,*N*′-Bis(acryloyl) cystamine (BAC) and acrylic acid (AAc) [16] with the HA-binding peptide GAH to mimic aggrecan function and generate an HA-binding nanoparticle (GAH-hNP). NIPAm is a thermoresponsive monomer with a lower critical solution temperature (LCST) of 32 °C, which, when polymerized into the particles, causes them to swell at temperatures below the LCST and constrict at temperatures above the LCST. AMPS is a highly sulfated, anionic monomer incorporated into the nanoparticles to mimic the charge provided by the GAGs attached to aggrecan. Moreover, the negative charge of AMPS maintains the colloidal stability of the nanoparticle. The incorporation of the homobifunctional degradable crosslinker BAC allows for particle degradation [16]. Finally, AAc serves as the carboxylate anchoring point within the hNP shell for peptide conjugation. These data presented here support the hypothesis that anionic hNP conjugated with GAH will restore the compressive stiffness of aggrecan-depleted cartilage and inhibit further degradation of its ECM. Further, nanoparticles are retained within the joint for at least 7 days. The detailed representation of the study is summarized in Figure 1.

## 2. Materials and Methods

### 2.1. Materials

*N*-isopropyl acrylamide (NIPAm, ≥98%, Cat. No. 415324), *N*,*N*′-Bis(acryloyl) cystamine (99%, BAC, Cat. No. A4929), *N*,*N*′-methylene-bis-diacrylaminde (97%, MBA, Cat. No. 377325), sodium dodecyl sulfate (SDS; 20% *w/v* in water, Cat. No. 436143), 2-acrylamido-2-methyl-1-propanesulfonic acid (99%, AMPS, Cat. No. 282731), Rhodamine B isothiocyanate (98%, RBITC, Cat. No. 23591-1), *N*-diisopropylethylamine (99%, DIPEA, Cat. No. BP592), potassium persulfate (99%, KPS, Cat. No. 216224), trifluoroacetic acid (TFA, Cat. No. BP618), 4-(4,6-Dimethoxy-1,3,5-triazin-2-yl)-4-methylmorpholinium chloride (96% DMTMM, Cat. No. H26333), dimethyl sulfoxide (DMSO, Cat. No. D2650), and porcine trypsin (Cat. No. T4799) were acquired from Sigma Aldrich (St. Louis, MO, USA). Dimethylformamide (DMF, Cat. No. BP1160-5), dichloromethane (DCM, BDH23373), acetonitrile (ACN, Cat. No. A998SK-4), triisopropylsilane (TIPS, AC214920500) and phenol (Cat. No 17914) were purchased from Thermo Fisher (Waltham, MA, USA). GAHWQFNALTVRGSG-Hydrazide (GAH-Hyd) was purchased from the Chinese Peptide Company (CPC, Hangzhou, China). Dialysis membrane tubing (Cat. No. 888-11023-H26) and tangential flow filtration carbon tubing (Cat. No. D02-E010-10-N) were purchased from Spectrum Laboratories (Dominguez, CA, USA). NIPAm and BAC were stored under nitrogen at 4 °C and −20 °C, respectively. AMPS was stored at room temperature in a desiccator. All water used in synthesis, dialysis, and testing was treated by a Millipore milliQ system (Billerica, MA, USA; 18.2 MΩ·cm resistivity). A full list of abbreviations is found in the Abbreviations section.

### 2.2. Nanoparticle Synthesis

The nanoparticle (NP) core–shell complex was polymerized via precipitation reaction as previously described [16]. Briefly, the NP cores were synthesized by dissolving 394.5 mg NIPAm in 3 mL milliQ water and injecting it into a 100 mL three-neck flask under reflux and a nitrogen blanket with 35 mL milliQ water and 164 µL of a 20% SDS solution at 70 °C. Following a 15 min equilibration time, 67.4 mg KPS dissolved in 2 mL milliQ water was injected into the reaction flask and continued for 2 h. NP cores were exposed to atmospheric oxygen for 45 min followed by a 15 min nitrogen purge. The NP shells were polymerized around the cores by injection of 794.7 mg NIPAm, 78.0 mg AMPS, 48.2 mg BAC, 4.81 µL AAc, and 164 µL 20% SDS dissolved in 5 mL milliQ water into the reaction flask. After 15 min, 33.7 mg KPS dissolved in 2 mL milliQ water was injected into the reaction flask, and the mixture was refluxed at 70 °C for 4 h. The nanoparticle solution was dialyzed in 10 kDa dialysis tubing (Spectrum Laboratories, Dominguez, CA, USA) at 4 °C for 14 days; milliQ water was changed daily. Following dialysis, the now-hollow NPs (hNPs) were frozen and lyophilized.

Fluorophore Incorporation: For RBITC-shell NP batches, 0.1 mol% RBITC dissolved in 1 mL DMSO was injected following NIPAm, AMPS, BAC, AAc, and SDS addition and before shell polymerization initiation, resulting in a fluorescently labeled co-poly(NIPAm-AMPS-AAc-BAC-RBITC) (hNPsRBITC) shell. Each NP batch was synthesized three times for experimental replicates and tested three times for technical replicates and placed in opaque coverings during dialysis and lyophilization to prevent photobleaching.

### 2.3. Peptide Synthesis

The majority of GAHWQFNALTVRGSG-Hydrazide (GAH-Hyd) was purchased from CPC while a portion was synthesized using the 2-Chlorotrityl Chloride (2-Cl-Trt) resin. Briefly, 2-Cl-Trt (1000 mg) was dissolved in DMF and washed in DMF, DCM, and DMF three times each. The hydrazide (0.5 mL) was dissolved with 100 µL DIPEA and 4.5 mL DMF and reacted for 2 h. To maximize hydrazide conjugation, this process was repeated. Glycine was added to the hydrazide by dissolving 1848.6 mg FMOC-Glycine and 875.6 mg OymaPure (Sigma Aldrich, St. Louis, MO, USA) in 5 mL DMF, added to the reaction vessel with 960 µL DIC plus 1060 µL DIPEA, and reacted overnight. This process was repeated to maximize glycine conjugation. The 2-Cl-Trt-Hyd-Gly resin was washed with DMF, DCM, and DMF three times each and then loaded into a CEM Liberty Blue Peptide Synthesizer (Matthews, NC, USA) to complete the peptide synthesis. Briefly, FMOC-protected L-amino acids were individually dissolved in synthesis-grade DMF to yield 0.2 M solutions. Synthesis occurred at 90 °C for 4–30 min per amino acid, with time varying for each amino acid. GAH-Hyd was cleaved from the 2-Cl-Trt resin using 2 mL of a cleavage cocktail (4.4 mL TFA, 0.25 mL phenol, 0.25 mL milliQ water, and 0.10 mL TIPS) for 3 h, precipitated with 0 °C diethyl ether, centrifuged at 1000× *g* for 5 min four times, and dried overnight at room temperature. GAH-Hyd was purified using reverse-phase fast-protein liquid chromatography (FPLC) (GE Healthcare AKTA, Chicago, IL, USA). Quantification of molecular weight was assessed using Matrix-Assisted Laser Desorption/Ionization—Time of Flight (MALDI-TOF) (Bruker, Billercia, MA, USA) mass spectroscopy.

### 2.4. Peptide Conjugation

GAH-Hyd was conjugated to the surface of hNPs using DMTMM chemistries in MES buffer at pH 4.5. Briefly, 0.5 mL of DMTMM at 75 mg/mL was added to a solution of 1 mg/mL hNPs, titrated to pH 4.5, and allowed to activate carboxylate groups for 30 min. Following activation, GAH-Hyd was added to the solution on a 0, 0.5, 1, 2, 4, and 6 to 1 molar equivalent to AAc polymerized within the hNP shell and reacted while stirring for 60 h. Analogous chemistries were used for RBITC-labeled hNPs, but extended to 0, 0.5, 1, 2, 4, 6, 8, 10, and 12-to-1 Aac molar equivalent within the hNPsRBITC shell. These chemistries were repeated using the 1:1 ratio of GAH to Aac within the hNPs in the absence of DMTMM and reacted for 60 h to quantify potential GAH adsorption to the particle. Following the reaction, all batches were purified using KR2i tangential flow filtration (TFF) from Spectrum Labs equipped with a 10 kDa nanofiber filter.

Conjugation was quantified using the Pierce Quantitative Peptide Colorimetric Assay (Thermofischer, Waltham, MA, USA) following manufacturer protocols. Briefly, 20 µL of each sample or standard was added to a 96-well clear bottom plate with 180 µL of the working reagent, incubated for 30 min at 25 °C, then the absorbance of each well was read at 480 nm using a Spectramax M5. A GAH peptide standard curve was used to calculate the peptide concentration.

### 2.5. Nanoparticle Characterization

Following purification and lyophilization, hNPs were dissolved at 1 mg/mL in milliQ water and subjected to temperature sweeps from 18.0 to 42.0 °C, in 1.5 °C increments, equilibrating for 3 min between each step, and measuring three times per step using dynamic light scattering (DLS) to assess the diameter and polydispersity index (PDI). The same procedure was followed after peptide conjugation to obtain their physical characteristics. The Zeta (ζ)-potential was obtained on a Nano-ZS90 Zetasizer at a 1 mg/mL sample concentration in milliQ water at 18.0 °C and 42.0 °C using folded capillary cells. All temperature trends and ζ-potential measurements were run in experimental and technical triplicate. The mass of the nanoparticle was calculated using the particle diameter assuming a density of 1 g/cm^3^. The mass of the nanoparticle and GAH quantification were used to calculate the amount of peptide per particle.

### 2.6. Dynamic Viscosity

Dynamic viscosities were measured on the Discovery HR-3 rheometer (TA Instruments, New Castle, DE, USA) set to flow sweep, equipped with a 20 mm stainless-steel plate with constant angular momentum and the temperature set to 37 °C. All therapeutics were dissolved in PBS then added to a solution of 700 kDA HA. Samples at 2.5 wt% HA were allowed to equilibrate to 37 °C for 3 min prior to testing. All samples underwent a shear sweep from 0.01 to 100 Hz. Dynamic viscosities were calculated using the slope of the shear rate values versus stress, based on a linear fit model. 

### 2.7. Tissue Harvest

Fetal bovine knees were purchased from Animal Technologies (Tyler, TX, USA) and cartilage explants were harvested 24 h after slaughter as previously described [17]. Briefly, using a cork borer, 3 mm diameter cartilage explants were taken from the load-bearing femoral condyle and washed three times with 1× PBS. Cartilage explants were then added to 25 mL of Dulbecco’s Modified Eagle Media (FBS DMEM)/F12 containing 0.1% bovine serum albumin, 100 units/mL penicillin, 100 µg streptomycin, and 3% FBS), then incubated at 37 °C for 10 min. Next explants were washed three times with Serum-Free DMEM/F12 then incubated in 400 µL 10% FBS DMEM/F12 in a 48-well plate for 3 days. 

### 2.8. Therapeutic Diffusion into Cartilage

Aggrecan was removed from cartilage explants using the previously described protocol [18]. Briefly, explants were washed three times with Hank’s Balanced Salt Solution (HBSS) then treated with 0.5% (*w/v*) trypsin in HBSS for 3 h at 37 °C. After treatment, explants were washed three times in HBSS and incubated within 20% FBS DMEM/F12 for 10 min to inactivate any remaining trypsin activity. Explants were treated with 10% FBS DMEM/F12 (Healthy) or 20 ng/mL IL-1β dissolved in 10% FBS DMEM/F12 to perpetuate inflammation for aggrecan-depleted (AD) samples. Therapeutics were dissolved in PBS to create a 1.6 mg/mL solution and 10 µL was placed on the surface of cartilage explants every 10 min for 1 h at room temperature. For penetration studies, hNPsRBITC or 20 GAH-hNPsRBITC was added to the top of the explant at 10 µL every 10 min for 1 h. For diffusion studies, 60 µL of a 1.6 mg/mL solution of 20 GAH-hNPsRBITC was placed on the top of the explant at time zero, and at 10, 30, 60, 120, 240, and 1440 min following the addition of the NP solution, the explant was removed, rinsed, and frozen in an optimal cutting temperature (OCT) compound. Following this, explants were cut in half, embedded in an O.C.T. compound (Tissue Tek), sectioned at 5 µm thickness using a cryostat (Leica 3050S, Leica Biosystems, Wetzlar, Germany), and imaged at 4× and/or 60× magnification using a Keyence Digital Microscope (Keyence, Osaka, Japan). Fick’s second law was used, assuming complete therapeutic diffusion at the 4 h timepoint and that the initial concentration was zero to obtain the diffusion constant:

$$\frac{C\left(x,t\right)-C_{0}}{C_{s}-C_{0}}=1-\mathrm{erf}\left(\frac{x}{2\sqrt{\mathrm{Dt}}}\right)\to C\left(x,t\right)=1-\mathrm{erf}\left(\frac{x}{2\sqrt{\mathrm{Dt}}}\right)$$

where C(x,t) is the concentration within the tissue at any given time, C_0_ is the initial concentration, t is the time, x is the depth, C_s_ is the solution concentration, and D is the diffusion coefficient.

### 2.9. Compression Testing

Cartilage explants were isolated and cultured as healthy (positive control), aggrecan-depleted (AD) (negative control), or AD and treated with unconjugated hNPs or 19 GAH-hNP. Explants were treated with 60 µL of 1.6 mg/mL (0.10 mg) and 6.4 mg/mL (0.38 mg) of unconjugated hNP or 19 GAH-hNP, respectively. The media was changed every two days and compressive stiffness was analyzed on day 0 for healthy explants only, and day 6 and day 12 for all other groups. Displacement-controlled unconfined compression was performed using a Discovery HR-3 rheometer (TA Instruments, New Castle, DE, USA). Explant height was measured (Duratool) and compressive loads were applied from 0 to 30% strain (at 5% intervals) with a 5 µm/s ramp and a hold time of 30 s. Moduli were calculated with the slope of the linear fit equilibrium stress vs. strain equation. Compression experiments were repeated twice with *n* = 5–7 per group per trial. 

### 2.10. GAG Quantification

Glycosaminoglycan degradation was measured by chondroitin sulfate (CS) release from the explant every 2 days in cell culture media using a dimethyl methylene blue (DMMB) assay [19,20]. The weight of the cartilage explant was recorded and CS release was reported as µg of CS released per mg of cartilage explant. 

### 2.11. Histology & Immunohistochemistry Assessment 

Cartilage explants were sectioned using the Leica 3050s cryostat at 5 µm thickness. Sectioned and plated tissue samples were submitted to the UC Davis VMTH Anatomic Pathology Service—Histopathology Lab (Davis, CA, USA) for all staining. Aggrecan depletion was assessed using Safranin O and counter stained with Fast Green. Immunohistochemistry (IHC) was performed to stain for collagen II using an anti-collagen II antibody (ab34712, Abcam, Cambridge, UK). Samples were imaged at 4× magnification using the Keyence Digital Microscope. The staining was measured from tissue samples using the area coverage per sample with NIH ImageJ software. The average coverage area was quantified by converting fluorescent images to binary and extracting pixel counts at bins 0 and 255. The stains were quantified using the percent area of the cartilage samples using the following equation:

$$\%\;\mathrm{Area}=100*\left(\frac{\mathrm{Number}\;\mathrm{of}\;\mathrm{Fluorescent}\;\mathrm{Pixels}}{\mathrm{Total}\;\mathrm{Number}\;\mathrm{of}\;\mathrm{Pixels}\;\mathrm{within}\;\mathrm{Cartilage}\;\mathrm{Sample}}\right)$$

### 2.12. In Vivo Nanoparticle Retention

Three-month-old Fischer 344 rats were purchased from Charles River (Wilmington, MA, USA). Following accumulation, rats were anesthetized with isoflurane and the hair was removed from both rat knees. Rats were injected with 0.10 mg of 20 GAH-hNPsRBITC dissolved in PBS (*n* = 6) into their left joint space or PBS alone as a negative control in the right joint space as a non-fluorescent control (*n* = 6). Fluorescence was measured and quantified using the In Vivo Imaging System (IVIS) at the UC Davis Center for Molecular and Genomic Imaging (CMGI) (Davis, CA, USA) at 557 nm excitation and 623 nm emission. Images were taken immediately prior to injection, post injection, daily for 7 days, then immediately following sacrifice and dissection. Rats were euthanized using CO_2_ asphyxiation. Total radiance emission (TRE) was assessed within a region of interest (ROI). The ROI was a uniform circle of 1.1 cm^2^ used for all rats and was anatomically placed around the knee using a grayscale image and were unbiased by fluorescent signals in a blinded fashion.

### 2.13. Statistical Analysis

Statistical differences of GAH conjugated particles, dynamic viscosity, zeta-potential, PDI, CS release, and histology and immunohistochemistry quantification were assessed using One-Way ANOVA. Two-Way paired ANOVA was used to assess statistical differences amongst compressive stiffness for cartilage explants and Two-Way ANOVA for GAH retention in vivo. For all analysis, groups that share a letter are statistically analogous, and if the groups do not share a letter, this represents statistically significant differences from one another, with significance being *p* < 0.05.

## 3. Results

### 3.1. Peptide Conjugation & Characterization

**Figure 2:**
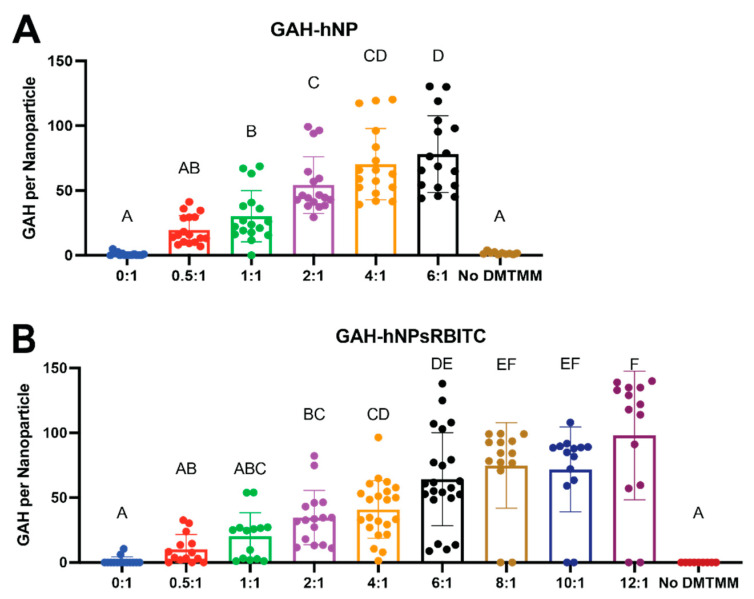
Increasing the molar ratio of GAH to AAc within hNP (**A**) or GAH to AAc within hNPsRBITC (**B**) increased the amount of GAH conjugated to the respective nanoparticles. Average values are summarized in Supplemental Tables S1 and S2. Different letters (A–F) denote statistically significant differences between groups while like letters represent groups that are statistically similar (*p* < 0.05).

The GAH-Hyd conjugation to the AAc polymerized into the hNPs and hNPsRBITCs was confirmed by the presence of a peptide on the particle, as shown in Figure 2. The increase in GAH concentration per mass of hNP indicated GAH attachment to both the hNP and hNPsRBITC. Nanoparticles incubated with GAH in the absence of DMTMM did not result in peptide conjugation to the nanoparticle as determined by results that showed only minor adsorption readings, indicative of the peptide, following NP purification. The average number of GAH peptides per nanoparticle is summarized in Table 1. The nomenclature subsequently used to describe the various groups tested was based on the number of peptides added per hNP. For example, 0.5:1 GAH to hNP reaction yielded roughly 19 GAH per hNP and is termed 19 GAH-hNP.

**Figure 3:**
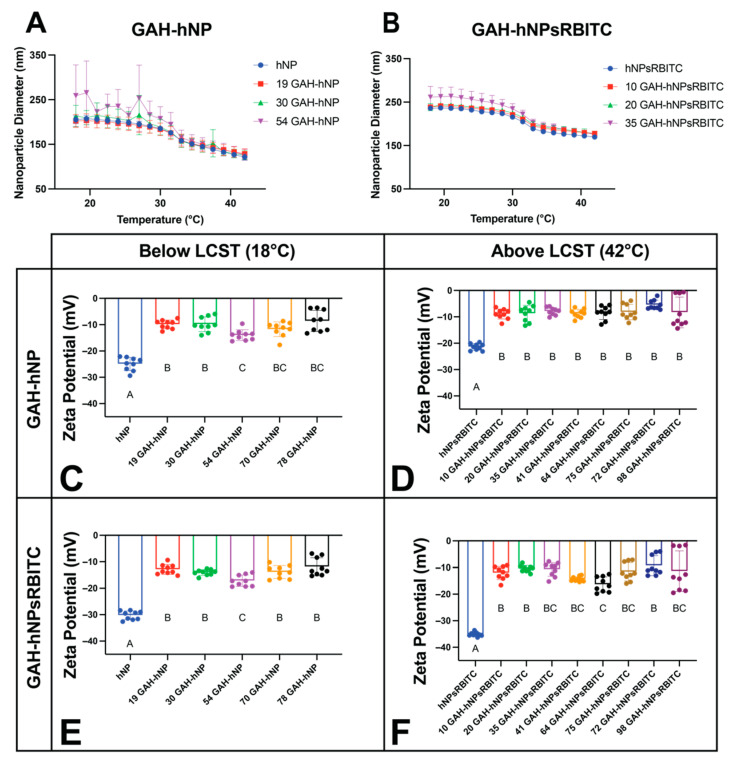
The diameter of the hNP (**A**) or hNPsRBITC (**B**) particles increased with increasing conjugation of GAH to the nanoparticle. Conjugating GAH to hNPs significantly increased the surface charge of the particles compared to unconjugated particles, below (**C**) and above (**D**) the LCST of pNIPAm. Conjugation of GAH to hNPsRBITC also increased the surface charge below (**E**) and above (**F**) the LCST of pNIPAm. Values are listed in Supplemental Tables S1 and S2. Different letters (A–C) denote statistically significant differences between groups while like letters represent groups that are statistically similar (*p* < 0.05).

Dynamic light scattering (DLS) confirmed nanoparticle diameter and thermoresponsive behavior of hNP and hNPsRBITC with increasing GAH conjugation, as shown in Figure 3A,B and Supplemental Figure S1. The unconjugated hNP and hNPs with 19–54 GAH had analogous diameters, as shown in Figure 3A. The same trend was observed with unconjugated hNPsRBITCs and hNPsRBITC conjugated with 20–35 GAH. The unconjugated hNPs, 70 GAH-hNP, and 78 GAH-hNPs had a diameter of 205.20 ± 8.83, 258.99 ± 69.16, and 986.36 ± 741.27 nm, respectively at 18.0 °C and 121.57 ± 6.71, 126.60 ± 12.27, and 182.38 ± 45.18 nm at 42.0 °C, respectively (Figure 3A and Supplemental Table S1). Notably, more than 41 GAH on the hNP and hNPsRBITC increased the polydispersity of the particles (Figure 3A,B and Supplemental Tables S1 and S2). The 78 GAH-hNP had a 4.58-times higher polydispersity index (PDI) than 19 GAH-hNP at 18.0 °C, and 98 GAH-hNPsRBITC had a 10.63-times higher PDI than 10 GAH-hNPsRBITC at 18.0 °C (Supplemental Figure S2 and Supplemental Tables S1 and S2). 

Increasing the conjugation of GAH to both hNP and hNPsRBITC significantly increased the zeta-potential of the particles. At 18.0 °C, unconjugated hNPs had a zeta-potential of −24.93 ± 2.53 mV, and peptide conjugation to the nanoparticle resulted in a roughly 2-fold increase in zeta-potential (Figure 3C and Supplemental Tables S1 and S2). The unconjugated hNPsRBITCs at 18.0 °C had a zeta-potential of −21.41 ± 1.26 mV at 18.0 °C and GAH conjugation resulted in a 23.72 to 56.53% increase in surface charge (Figure 3E and Supplemental Tables S1 and S2).

### 3.2. Hyaluronic Acid Binding and Diffusion into Cartilage Explants

**Figure 4:**
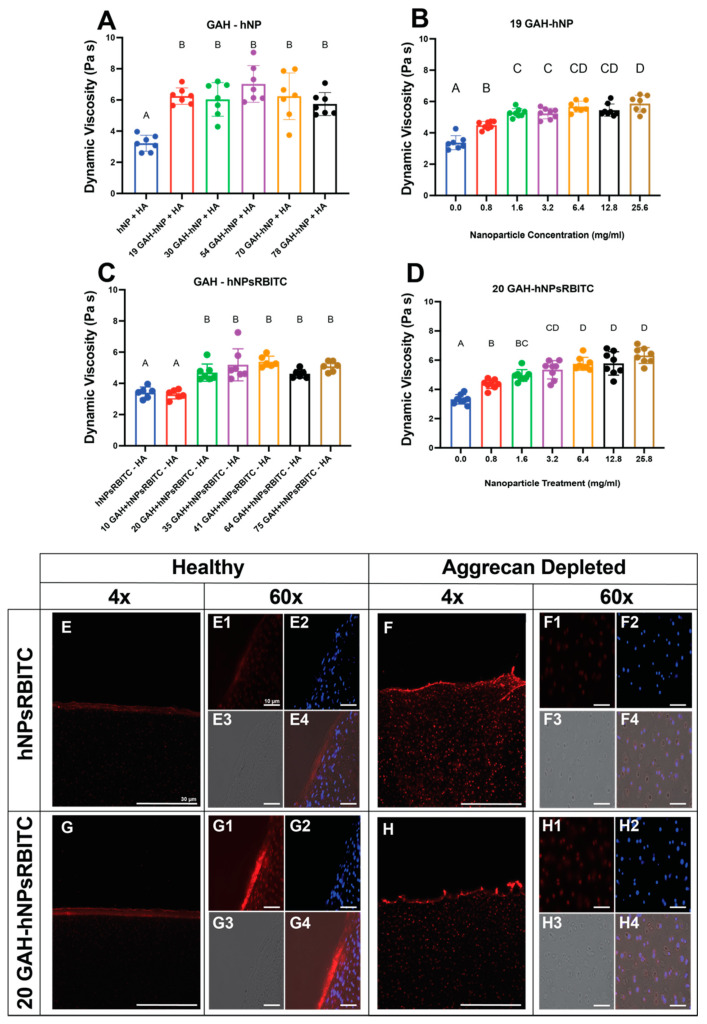
(**A**–**D**) Dynamic viscosity (DV) of the HA solution by GAH-hNP or GAH-hNPsRBITC and (**E**–**H**) diffusion of unconjugated hNPsRBITC or 20 GAH-hNPsRBITC into (**E**,**G**) healthy or (**F**,**H**) aggrecan-depleted cartilage explants. (**A**) GAH-hNP significantly increased DV at varying peptide/nanoparticle conjugations. (**B**) Increasing 19 GAH-hNP concentration within the HA solution increased DV. (**C**,**D**) Increasing the concentration of 19 GAH-hNP increased DV of the HA solution. (**E**–**H**) Sagittal cross section of load-bearing fetal bovine articular cartilage. Healthy (**E**,**G**) and aggrecan-depleted (**F**,**H**) ex vivo cartilage plugs treated with unconjugated hNPsRBITC (**E**,**F**) and 20 GAH-hNPsRBITC (**G**,**H**). Unconjugated hNPsRBITC and 20 GAH-hNPsRBITC significantly penetrated into aggrecan-depleted cartilage. (**E1**–**H1**): RBITC; (**E2**–**H2**): Hoechst (Nuclei); (**E3**–**H3**): Brightfield; (**E4**–**H4**): Overlay. Scale bar for (**A**–**H**): 30 µm; ((**E1**–**H1**)–(**E4**–**H4**)): 10 µm. Different letters (A–D) denote statistically significant differences between groups while like letters represent groups that are statistically similar (*p* < 0.05).

The GAH-hNP and GAH-hNPsRBITC particles bind to HA as measured by the increase in dynamic viscosity (DV) of a free HA solution treated with GAH-hNP and GAH-hNPsRBITC particles (Figure 4A,C). The DV of the HA solution treated with unconjugated hNPs and hNPsRBITC was 3.2 ± 0.5 Pa.s and 3.4 ± 0.4 Pa.s, respectively, and 19 GAH-hNP and 20 GAH-hNPsRBITC had a DV of 6.2 ± 0.5 Pa.s and at 4.8 ± 0.6 Pa.s, respectively. In comparison, 10 GAH-hNPsRBITC had a 3.4% increase in DV compared to unconjugated hNPsRBITC and did not elicit significant HA binding, as shown in Figure 4C. All other groups had analogous increases in DV to the 19 GAH-hNP and 20 GAH-hNPsRBITC when compared to their unconjugated hNP or unconjugated hNPsRBITC, as can be seen in Figure 4A,C. hNPs and hNPsRBITCs conjugated with 19–35 GAH remained monodisperse and significantly bound to HA (Figure 4A,C, Supplemental Tables S1 and S2). We subsequently proceeded with particles conjugated with 19 GAH-hNP and 20 GAH-hNPsRBITC since they elicited statistically similar increases in DV compared to the respective unconjugated nanoparticles and were monodisperse.

As determined by DV measurements, all concentrations of 19 GAH-hNP and 20 GAH-hNPsRBITC are significantly bound to HA (Figure 4B,D). Notably, treatment with 60 µL of 3.2, 6.4, 12.8, and 25.6 mg/mL of 19 GAH-hNP and 20 GAH-hNPsRBITC showed similar HA binding and had at least a 54.6% increase in DV compared to their respective controls, as shown in Figure 4B,D. The treatment with 60 µL of 0.8 mg/mL 19 GAH-hNP and 20 GAH-hNPsRBITC had a 28.4% and 31.7% increase in DV compared to the control, respectively. Treatment with 60 µL of 1.6 mg/mL 19 GAH-hNP and 20 GAH-hNPsRBITC had a 54.4% and 49.2% increase in DV compared to the PBS control, respectively, as can be seen in Figure 4B,D. The subsequent ex vivo cartilage explant studies proceeded with treatment with 60 µL of 1.6 mg/mL (0.10 mg) of 19 GAH-hNP or 0.10 mg of 20 GAH-hNPsRBITC as the lower mass per cartilage plug, and 60 µL of 6.4 mg/mL (0.38 mg) of 19 GAH-hNP or 0.38 mg of 20 GAH-hNPsRBITC as the higher mass per cartilage plug since they represent the high and low values of the measured effective HA-binding.

### 3.3. Diffusion into Aggrecan Depleted Cartilage Explants 

After 48 h of incubation with unconjugated hNPsRBITC and 20 GAH-hNPsRBITC, fluorescent images of cryosectioned tissue showed that hNPsRBITC and 20 GAH-hNPsRBITC remained on the surface of healthy articular cartilage, as shown in Figure 4E,G, while hNPsRBITC and 20 GAH-hNPsRBITC permeated into AD cartilage explants, as shown in Figure 4F,H. Figure 4(F4,H4) show the overlay of hNPsRBITC and 20 GAH-hNPsRBITC, respectively, with nuclei of chondrocytes within the explants suggesting localization of the particles near chondrocytes. Moreover, roughly 4 h was required for unconjugated hNPsRBITC and 20 GAH-hNPsRBITC to significantly diffuse into the AD explant (Supplemental Figure S3). The diffusion coefficient of the 20 GAH-hNP into the AD explant was 6.5 µm^2^/s [21].

### 3.4. Compression Testing

**Figure 5:**
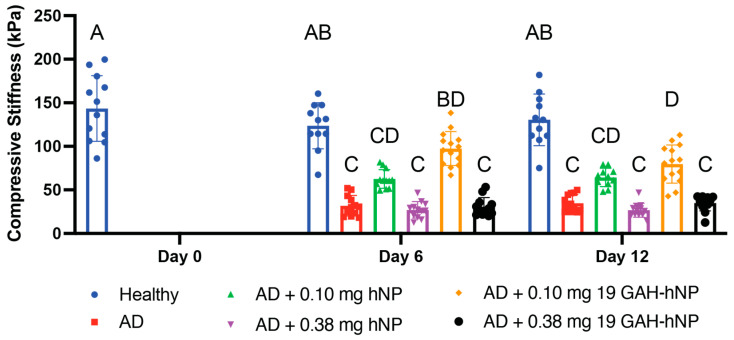
Treatment with 0.10 mg of 19 GAH-hNPs significantly restored the compressive stiffness of osteoarthritic ex vivo cartilage explants at day 6 and day 12. Data are represented as mean ± StDev (*n* = 10–12 per treatment per timepoint). Different letters (A–D) denote statistically significant differences between groups while like letters represent groups that are statistically similar (*p* < 0.05).

The untreated-healthy explants and untreated-AD cartilage explants had a compressive stiffness of 122.1 ± 26.6 kPa and 31.9 ± 11.8 kPa on day 6, and 130.5 ± 29.7 kPa and 34.8 ± 9.9 kPa on day 12, respectively. The AD explants showed a 73.8% and 73.3% loss in compressive stiffness on days 6 and 12, respectively, compared to untreated-healthy controls on the same day (Figure 5). The AD explants treated with 0.10 mg of 19 GAH-hNP had a compressive stiffness of 95.6 ± 16.2 kPa on day 6 and were statistically analogous to untreated-healthy explants, as shown in Figure 5. The AD explants treated with 0.10 mg of 19 GAH-hNP on day 12 had a compressive stiffness of 79.7 ± 21.8 kPa and were statistically similar to explants treated with 0.10 mg of 19 GAH-hNP on day 6 but had a 38.9% loss in compressive stiffness compared to untreated-healthy explants on the same day, as can be seen in Figure 5. The untreated-AD explants and explants treated with 0.10 mg of hNP had statistically analogous compressive stiffness on day 6 and day 12. However, they had a compressive stiffness of 63.3 ± 10.5 kPa and 64.3 ± 10.5 kPa on day 6 and day 12, respectively—a 198.8% and 184.8% increase in compressive stiffness compared to untreated-AD explants on the same day (Figure 5). The AD explants treated with 0.38 mg of hNP and 0.38 mg of 19 GAH-hNP had similar compressive stiffness, 27.0 ± 9.4 kPa and 30.6 ± 10.6 kPa on day 6 and 26.6 ± 8.0 kPa and 34.8 ± 8.6 kPa on day 12, respectively, to untreated-AD explants (Figure 5). 

### 3.5. ECM Degradation

**Figure 6:**
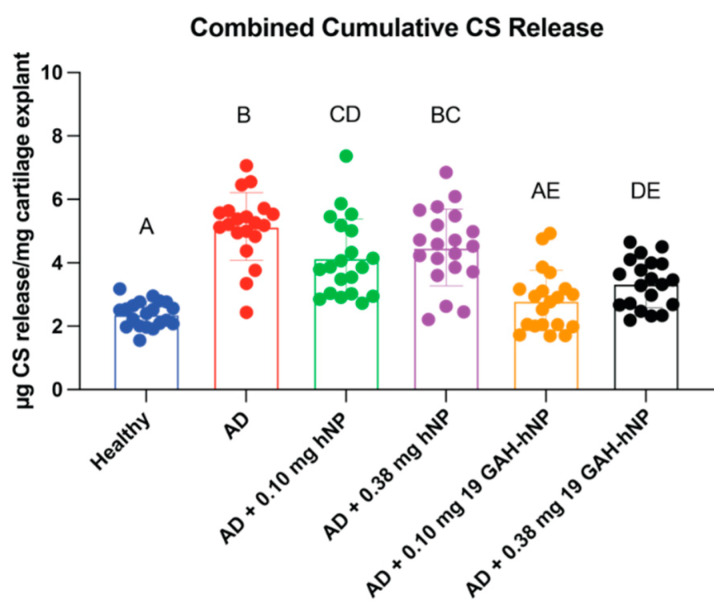
Treatment with 0.10 mg of 19 GAH-hNP inhibited further degradation of the ECM of AD cartilage as quantified by accumulative CS release. Different letters (A–E) denote statistically significant differences between groups while like letters represent groups that are statistically similar (*p* < 0.05).

The amount of CS released from explants treated with 0.10 mg of 19 GAH-hNP was statistically similar to healthy cartilage (Figure 6). The explants treated with 0.38 mg of 19 GAH-hNP released 18.19% more CS than explants treated with 0.10 mg of 19 GAH-hNP. Untreated-AD explants and explants treated with 0.10 mg and 0.38 mg of hNPs had a 53.94%, 43.96%, and 47.81% increase in CS release, respectively, compared to untreated-healthy explants (Figure 6). However, explants treated with 0.10 mg of hNP had a 17.78% decrease in CS release compared to untreated-AD explants. There was no significant difference in CS release of the explants treated with 0.38 mg of hNP compared to untreated-AD explants, as shown in Figure 6. Notably, the DMMB assay did not react with the sulfated hNPs, demonstrating that the DMMB signal is associated with GAGs (Supplemental Figure S5).

### 3.6. Histology and Immunohistochemistry

**Figure 7:**
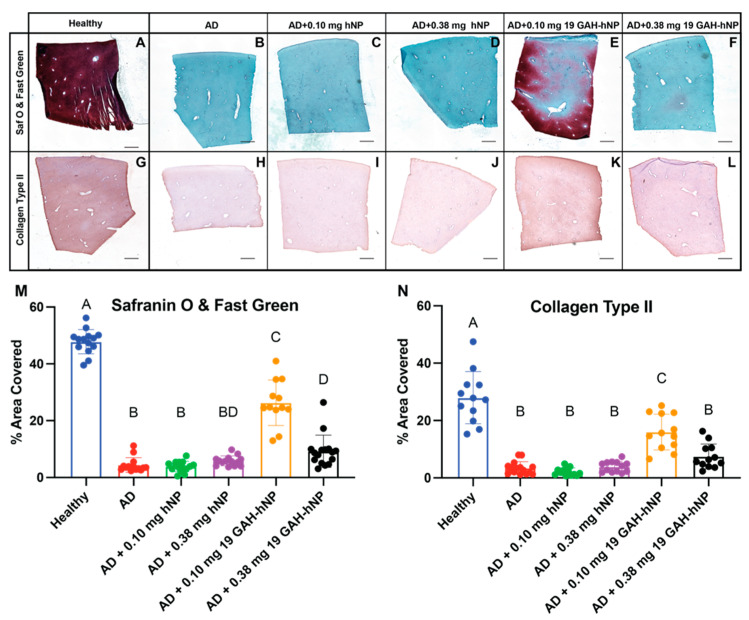
Safranin O and Fast Green staining of cartilage explants to quantify GAG content (**A**–**F**). IHC using the anti-collagen II antibody (**G**–**L**). The explants treated with 0.10 mg of 19 GAH-hNP (**E**,**K**) inhibited the degradation of the ECM. Scale bar 100 µm. (**A**,**G**) untreated-healthy explant; (**B**,**H**) untreated-AD explant; (**C**,**I**) AD explant treated with 0.10 mg hNP; (**D**,**J**) AD explant treated with 0.38 mg hNP; (**E**,**K**) AD explant treated with 0.10 mg 19 GAH-hNP; (**F**,**L**) AD explant treated with 0.38 mg 19 GAH-hNP. (**M**) Area percent covered of Safranin O and Fast Green stain within cartilage explants. (**N**) Area percent covered of collagen type II IHC within cartilage explants. Different letters (A–D) denote statistically significant differences between groups while like letters represent groups that are statistically similar (*p* < 0.05).

Positive Safranin O staining showed the presence of GAGs in untreated-healthy explants and AD explants treated with 0.10 mg of 19 GAH-hNP, with some expression in explants treated with 0.38 mg of 19 GAH-hNP (Figure 7A,E,F,M). The explants treated with 0.10 mg of 19 GAH-hNP had a 44.95% decrease in GAG content, compared to untreated-healthy explants. However, AD-explant treated with 0.10 mg of 19 GAH-hNP had 5.99-times more GAG content than untreated-AD explants (Figure 7A,B,E,M). The untreated-AD explants and explants treated with 0.10 mg and 0.38 mg of hNP showed a significant loss of GAGs and were all statistically similar with respect to GAG content, as seen in Figure 7B–D,M. Notably, Safranin O and Fast Green does not stain the sulfated hNPsRBITC, shown in Supplemental Figure S4. 

The untreated-AD explants had an 87.58% loss of collagen type II, compared to untreated-healthy explants (Figure 7H,G,N, respectively). The explants treated with 0.10 mg of 19 GAH-hNP has 409.1% more collagen type II than untreated-AD explants (Figure 7K,H,N). However, the explants treated with 0.10 mg of 19 GAH-hNP had 49.19% less collagen type II than the untreated-healthy explants, as shown in Figure 7G,K,N. The explants treated with 0.10 mg and 0.38 mg of hNP showed a 92.39% and 86.15% loss of collagen type II compared to untreated-healthy explants (Figure 7I,J,N). The explants treated with 0.38 mg of 19 GAH-hNP showed a 73.25% loss in collagen type II compared to untreated-healthy explants (Figure 7H,L,N). 

### 3.7. Retention of GAH-hNPsRBITC within Joint Space

**Figure 8:**
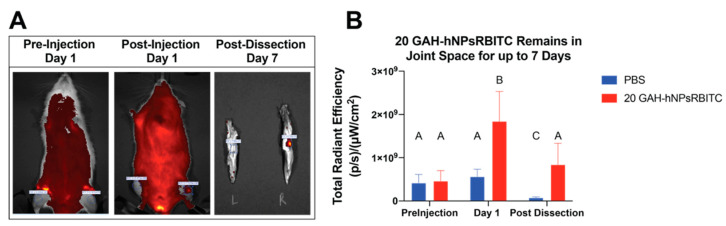
20 GAH-hNPsRBITC remains in the joint space for at least 7 days following injection. Different letters denote statistically significant differences between groups while like letters represent groups that are statistically similar (*p* < 0.05). (**A**) In vivo images of rat pre and post injection and dissected limbs 7 days after injection; (**B**) quantification of TRE; average TRE ± StDev. Different letters (A–C) denote statistically significant differences between groups while like letters represent groups that are statistically similar (*p* < 0.05).

20-GAH-hNPsRBITC was injected into and retained within the joint space of rats for at least 7 days as confirmed by the 400.1% increase in total radiant efficiency (TRE) of the injected knee compared to the same knee before injection (Figure 8). Moreover, 24 h after the injection, the 20 GAH-hNPsRBITC-injected knee had a 327.1% increase in TRE compared to the PBS-injected knee. After dissection, the 20 GAH-hNPsRBITC-injected knee had a 958.9% increase in TRE compared to the PBS injected knee, as shown in Figure 8. 

## 4. Discussion

Nanomedicine offers a potential solution to halt or even reverse the progression of PTOA. There are several polymeric [22,23,24,25,26], lipid [27,28,29,30,31] and metallic [32,33] nanotherapeutics currently being studied to treat OA. However, many emerging OA nanotherapeutics do not specifically target osteoarthritic cartilage nor do they inhibit the degradation of its ECM. They instead focus on inflammation and/or joint pain [22,23,24,25,26,27,28,29,30,32,33]. Modifying nanoparticles to bind to and treat damaged osteoarthritic cartilage offers a solution to this current limitation. Recent advances within nanomedicine have resulted in modified nanoparticles functionalized with antibodies for cell targeting [34], poly(ethylene glycol)(PEG) for increased biocompatibility [35,36], and peptides for cell targeting and as therapeutics [37]. Peptide-NPs are primarily used for biomarker detection and molecular imaging probes, with the nanoparticles used being mostly metallic [37,38,39]. Here, we build upon the targeting success of peptide-NPs where the HA-binding peptide GAH was conjugated to anionic hNPs to target damaged cartilage and support nanoparticle therapeutic function to inhibit the progression of OA.

Preventing aggrecan degradation within osteoarthritic cartilage is difficult since its loss happens quickly following joint trauma and inflammation. Therefore, we aimed to mimic the aggrecan function using an anionic nanoparticle conjugated with the peptide GAH. CS and keratin sulfate (KS) compose the anionic GAGs component of aggrecan and provide a net negative charge, thus allowing the aggrecan to generate an osmotic gradient that supports water retention and provides articular cartilage with its compressive stiffness [5]. To mimic the protective effects of aggrecan, we used our previously described anionic, degradable, poly(NIPAM-co-AMPS-AAc-BAC) hollow nanoparticles (hNPs) [16] and functionalized them with GAH to supporting HA-binding. 

While at low conjugation levels, the GAH peptide did not alter the collapsed and swollen diameters of the nanoparticles, high conjugation levels had a pronounced effect. Specifically, the addition of 35 or fewer GAH peptides to the nanoparticles did not significantly alter final nanoparticle diameter either below or above the LCST, while conjugation levels above 41 peptides did (Figure 3A,B and Supplemental Tables S1 and S2). This insight is critical since nanoparticles with a diameter over 200 nm are known to initiate an inflammatory response in vivo [40]. A PDI of less than 0.2 signifies monodisperse particles in solution [41]. It was further observed that the PDI of nanoparticles with 19 to 35 GAH was <0.2 while the nanoparticles with more than 41 GAH were polydisperse. The GAH peptide contains several hydrophobic amino acids including tryptophan, phenylalanine, leucine, and valine that could, when present at high concentrations on the particle surface such as with particles with greater than 41 GAH per particle, support particle agglomeration that increased the PDI (Supplemental Figures S1 and S2). The diameter and PDI data suggest that there is a maximum concentration of the peptide that can be conjugated to the particle before agglomeration occurs. PDI is crucial for the quality control of nanotherapies, and the FDA recently published guidelines for liposome (lipid-based nanoparticle) drug products emphasizing the importance of size and size distribution as “critical quality attributes (CQAs)” [42]. The 19 GAH-hNP fits the requirements of being both monodisperse and below 200 nm.

To verify that the GAH-hNPs are bound to HA, we used dynamic viscosity, a value that increases with apparent polymer molecular weight. Since GAH-hNP should crosslink HA, both the apparent molecular weight and dynamic viscosity should increase with increasing GAH-hNP added to the HA solution until maximal crosslinking occurs. The 19 GAH-hNPs and 20 GAH-hNPsRBITC increased in DV compared to an HA solution treated with unconjugated particles. However, greater conjugation of the peptide to the nanoparticles did not further increase dynamic viscosity. This result may be due to particle aggregation at high peptide conjugation density as described earlier or may indicate that additional peptides per nanoparticle do not increase crosslink formation. Examining the DLS and DV data together to find peptide conjugation densities that supported HA binding without inducing particle aggregation led to the use of 19 GAH-hNP and 20 GAH-hNPsRBITC for additional studies. 

Treating cartilage explants with trypsin has been shown to strip aggrecan from the explant without damaging chondrocytes, HA, or collagen, and serves as an ex vivo model for osteoarthritis [7,17,43,44]. Unconjugated hNPsRBITC and 20 GAH-hNPsRBITC permeated into aggrecan-depleted explants while they remained at the surface of the healthy explants. These data agreed with previous studies where anionic bottle-brush polymers and pNIAPm-based nanoparticles diffused into damaged cartilage [21,23], while pNIPAm-based nanoparticles remained on the surface of healthy cartilage [23]. The 20 GAH-hNPsRBITC diffused slower than the hNPsRBITC, potentially due to differences in HA binding within the explant, though both particle types significantly permeated into AD cartilage after 4 h.

Previously, GAH was conjugated to anionic CS and was shown to restore the compressive stiffness of aggrecan-depleted cartilage [7]. Here, we show that synthetic nanoparticles with a high anionic character also increased the compressive stiffness of damaged cartilage. The ability of 19 GAH-hNP to restore compressive stiffness and inhibit further ECM degradation was tested using cultured bovine explants. In other studies, healthy cartilage explants tested under unconfined compression had stiffness of 100–200 kPa, and damaged untreated cartilage had stiffness of 40–60 kPa, matching our data [7,45,46]. Stripping the explants of aggrecan resulted in >70% loss of compressive stiffness on days 6 and 12 compared to healthy controls, highlighting the importance of aggrecan to maintain the compressive stiffness within the joint [4]. While the compressive stiffness improved slightly with hNP, statistically significant improvement was only seen with GAH-hNPs, demonstrating the importance of HA-binding similar to that seen with aggrecan. The observed improvement in compressive stiffness is likely due largely to retention of the nanoparticles, and thus ionic charge, within the cartilage ECM; however, it is also likely that the GAH-hNPs serve as transient crosslinks within the damaged tissue that may also contribute to the compressive stiffness. The decrease in compressive stiffness observed between days 6 and 12 in GAH-hNP-treated cartilage suggests that some of the particles are lost from the tissue either via diffusion out of the tissue or by degradation of the particles over time. The colocalization of 20 GAH-hNPsRBITC with chondrocytes, shown using immunofluorescent imaging in Figure 4, suggests some particle endocytosis. Previously, the poly(NIPAm-co-AMPS-AAc-BAC) hNPs were shown to be endocytosed, degraded, and cleared from chondrocytes in 5–7 days in vitro [16]. Interactions with HA may dampen the uptake of 19 GAH-hNP by chondrocytes and slow their endosomal degradation, but it is unlikely that cell uptake is eliminated. 

Further, glutathione (GSH) is present in the ECM, although at lower concentrations than that within the cells [47,48], and can degrade the disulfate crosslinked GAH-hNPs even in the ECM. The unconjugated hNPs can both be more readily endocytosed and degraded and diffuse from the tissue, which would account for both their loss and a decrease in the restored compressive stiffness. Together, these data suggest that nanoparticle degradation and/or diffusion from the tissue occurs, and this loss of particles from the tissue happens more quickly than the deposition of new aggrecan. This is especially true for unconjugated hNPs. Notably, the explants treated with 0.38 mg showed no restoration of compressive stiffness. This is believed to be due to a rapid buildup of the particles on the surface of the cartilage at the time of treatment, which formed an anionic particle layer that inhibited therapeutic diffusion into the AD explant. This has been similarly observed in a previous study using anionic polymer-treated AD explants [43].

Osteoarthritis results in the progressive loss of matrix from cartilage tissue. Nominal CS release is normal from healthy cartilage, while excess CS release leads to increased catabolic enzyme expression and the irreparable degradation of collagen type II—resulting in OA [7,15]. Data collected from AD-explants cultured in the presence of inflammatory cytokines to further mimic OA conditions and treated with 0.10 mg 19 GAH-hNP showed dampened CS release as compared to untreated-AD explants. To verify that we assessed CS release and not the release of sulfated nanoparticles, we validated that DMMB does not bind to and detect the nanoparticles (Supplemental Figure S5). DMMB binds to a sulfated tetrasaccharide sequence in GAGs, so this finding was anticipated [20]. We further validated that treatment with 19 GAH-hNPs slowed the ECM degradation of articular cartilage under inflammation using immunohistochemistry. Immunohistochemistry showed the presence of both collagen type II and GAG in the ECM of AD-cartilage that was treated with 0.10 mg of 19-GAH-hNP (Figure 7). We verified that Safranin O did not stain the sulfated nanoparticles within AD tissue to ensure that the staining observed indeed came from GAG present within the tissue (Supplement Figure S4). Both fragmented HA and collagen are known catabolic stimulants, therefore dampening the degradation of these components may help slow OA progression [49]. In addition to limiting matrix degradation and the production of catabolic fragments of collagen and HA, the GAH-hNPs may act to sterically hinder catabolic enzyme diffusion into the matrix and inhibit HA and collagen degradation, thus protecting the ECM. The composition and structure of the ECM of cartilage are crucial for chondrocyte homeostasis. Chondrocytes have a low cell density within cartilage, and direct cell-to-cell communication is difficult. The ECM of cartilage has been shown to transmit mechanical signals between chondrocytes. Irreplaceable collagen loss associated with the progression of OA would affect chondrocyte-to-chondrocyte communication [50]. While there was a loss of GAGs and collagen type II compared to untreated-healthy explants, these data show the 19 GAH-hNP slowed the degradation of the ECM of articular cartilage in AD cartilage explants.

Current intra-articular delivery of free therapeutics showed less than a 3-day therapeutic retention time within the joint [51,52]. We examined the retention of 19 GAH-hNP in a rat joint. HA is a major component of the synovial fluid and is turned over roughly every 13 h [53], so binding to HA could reduce GAH-conjugated nanoparticles’ joint retention time. The GAH-conjugated particles remained in the joint for up to 7 days, as shown in Figure 8. Previously, we showed that unconjugated hNPsRBITC injected into the joint space had no loss of TRE between the initial injection and at the conclusion of 7 days [16]. Here, the concentration of 19 GAH-hNP was approximately 50% lower than that seen one day following injection. This decrease may be due to particle clearance with the synovial fluid or degradation of the particles as discussed previously. However, even with a 50% reduction in particle concentration, the particles exceed small-molecule joint retention time. Future studies will investigate catabolic enzyme secretion and the dosage of NPs needed to observe a therapeutic effect in vivo, as well as the major metabolic pathway of GAH-hNP within the joint. In addition, these nanoparticles have been shown previously to load and release therapeutic doses of an anti-inflammatory MAPKAP Kinase 2 (MK2) inhibitor peptide [16]. Future studies will investigate the potential therapeutic benefit of combining the protective effects conferred by these anionic, HA-binding particles with the controlled release of anti-inflammatory, MK2 inhibitor peptides.

## 5. Conclusions

Here we highlight the ability of hollow, degradable nanoparticles to be functionalized with ECM-binding peptides using DMTMM peptide coupling chemistry. Increasing the molar equivalent of the GAH-peptide to AAc polymerized into the poly(NIPAM-co-AMPS-AAc-BAC) and poly(NIPAM-co-AMPS-AAc-BAC-RBITC) particles led to increased peptide concentration on the hNPs and hNPsRBITCs, respectively. Notably, increasing the peptide amount to more than 41 GAH per particle led to increased polydispersity. In ex vivo cartilage studies, AD explants treated with 0.10 mg of 19 GAH-hNP showed restored compressive stiffness and inhibited ECM degradation. Finally, the 19 GAH-hNP therapeutic was retained within the joint space of rats for 7 days. GAH-hNP could be used clinically to treat patients diagnosed with early-stage OA. GAH-hNP treatment may reduce further ECM degradation and promote GAG synthesis, thereby treating and preventing the progression of OA. Treatment with 0.10 mg of 19 GAH-hNP showed promise in inhibiting the degradation of cartilage associated with OA. 

## Figures and Tables

**Table 1:** The average number of GAH peptides per nanoparticle.

GAH to AAc Ratio	GAH/hNP	GAH/hNPsRBITC
0:1	0	0
0.5:1	19	10
1:1	30	20
2:1	54	35
4:1	70	41
6:1	78	64
8:1	N/A	75
10:1	N/A	71
12:1	N/A	98
No DMTMM	0	0

## Data Availability

The data presented in this paper can be found at doi:10.25338/B8FS8G.

## Supplementary Materials

The following are available online at https://www.mdpi.com/article/10.3390/pharmaceutics13091503/s1, Figure S1: Increasing peptide concentration on the surface of hNP and hNPsRBITC significantly increases the variation in nanoparticle diameter, Table S1: GAH-hNP diameter, PDI, and Zeta-Potential at increasing GAH concentration on the surface of hNP, Table S2: GAH-hNPsRBITC diameter, PDI, and Zeta-Potential at increasing GAH concentration on the surface of hNPsRBITC, Figure S2: Increasing GAH concentration on the surface of hNP and hNPsRBITC increasing polydispersity of particles in solution. Direct values listed in Supplemental Tables S1 and S2. Figure S3: Timed diffusion of 20 GAH-hNPsRBITC into AD cartilage explants, Figure S4: Aggrecan-depleted explants treated with 0.10 mg unconjugated hNPsRBITC, frozen in OCT, and sectioned. The explants were quantified for hNPsRBITC (A) and were stained with Safranin O and Fast Green (B) to assess whether the sulfated AMPS within the hNPsRBITC were stained as well. The Safranin O and Fast Green stain does not stain the hNPsRBITC. Scale bars are 1000 µm, Figure S5: Standard curves of chondroitin sulfate (CS) (red) and hNP (blue) using DMMB assay.

## Abbreviations

AAc	Acrylic Acid
ACI	Autologous Chondrocyte Implantation
ACN	Acetonitrile
AD	Aggrecan Depleted
AMPS	2-acrylamido-2-methyl-1-propanesulfonic acid
BAC	*N*,*N*′-bis (acryloyl) cystamine
CMGI	Center for Molecular and Genomic Imaging
CS	Chondroitin Sulfate
DCM	Dichloromethane
DIPEA	N-Diisopropylethylamine
DLS	Dynamic Light Scattering
DMEM	Dulbecco’s Modified Eagle Media
DMF	Dimethylformamide
DMSO	Dimethyl Sulfoxide
DTT	Dithiothreitol
ECM	Extracellular Matrix
FBS	Fetal Bovine Serum
FDA	Food and Drug Administration
FPLC	Fast-Protein Liquid Chromatography
GAG	Glycosaminoglycan
GAH	GAHWQFNALTVRGSG HA-binding Peptide
GSH	Glutathione
HA	Hyaluronic Acid
HBSS	Hank’s Balanced Salt Solution
hNP	Hollow Nanoparticle
HPLC	High-Performance Liquid Chromatography
IA	Intra-Articular
IGD	Interglobular Domain
IL-1β	Interleuken-1 Beta
IL-6	Interleuken-6
IVIS	In Vivo Image System
KPS	Potassium Persulfate
KS	Keratin Sulfate
LCST	Lower Critical Solution Temperature
MBA	*N*,*N*′-methylene-bis-diacrylaminde
MALDI-TOF	Matrix Assisted Laser Desorption/Ionization-Time of Flight
MAPKAP 2	Mitogen Activated Protein Kinase Activated Protein Kinase 2
MK2	MAPKAP2
MK2i	MK2 inhibitor
MMP	Matrix Metalloprotease
NIPAm	*N*-isopropylacrylamide
NP	Nanoparticle
NSAID	Non-Steroidal Anti-Inflammatory Drug
pNIPAm	Poly(*N*-isopropylacrylamide)
PDI	Polydispersity Index
PEG	poly(ethylene glycol)
PTOA	Post Traumatic Osteoarthritis
OA	Osteoarthritis
RBITC	Rhodamine B Isothiocyanate
SDS	Sodium Dodecyl Sulfate
TFA	Trifluoroacetic Acid
TFF	Trangential Flow Filtration
TIPS	Triisopropylsilane
TRE	Total Radiance Emission
