# Supplementary material for: Hyaluronic Acid-Binding, Anionic, Nanoparticles Inhibit ECM Degradation and Restore Compressive Stiffness in Aggrecan-Depleted Articular Cartilage Explants
Source: Pharmaceutics. 2021 Sep 18;13(9):1503. doi: 10.3390/pharmaceutics13091503 (PMC8469381; doi:10.3390/pharmaceutics13091503)
Supplement: Supplementary file 1 [file pharmaceutics-13-01503-s001.zip › pharmaceutics-1370056-supplementary.pdf]

pharmaceutics

MDPI

Supplementary Material

Hyaluronic Acid-Binding, Anionic, Nanoparticles Inhibit ECM Degradation and Restore Compressive Stiffness in Aggrecan-Depleted Articular Cartilage Explants

Marcus Deloney [1], Parssa Garoosi [1], Vanessa F.C.Dartora [1,2], Blaine A.Christiansen[3] and Alyssa Panitch [1,2,*]

[1] Biomedical Engineering Department, 451 E. Health Sciences Dr. Room 2303, University of California Davis, Davis, CA 95616, USA; madeloney@ucdavis.edu (M.D.); pdgaroosi@ucdavis.edu (P.G.); vfcdartora@ucdavis.edu (V.F.C.D.)

[2] Department of Surgery, School of Medicine, University of California Davis, Sacramento, CA 95817, USA

[3] Lawrence J. Ellison Musculoskeletal Research Center, Department of Orthopedic Surgery, University of California Davis Health, 4635 2nd Avenue, Suite 2000, Sacramento, CA 95817, USA; bchristiansen@ucdavis.edu

* Correspondence: apanitch@ucdavis.edu; Tel.: +1-530-754-3222

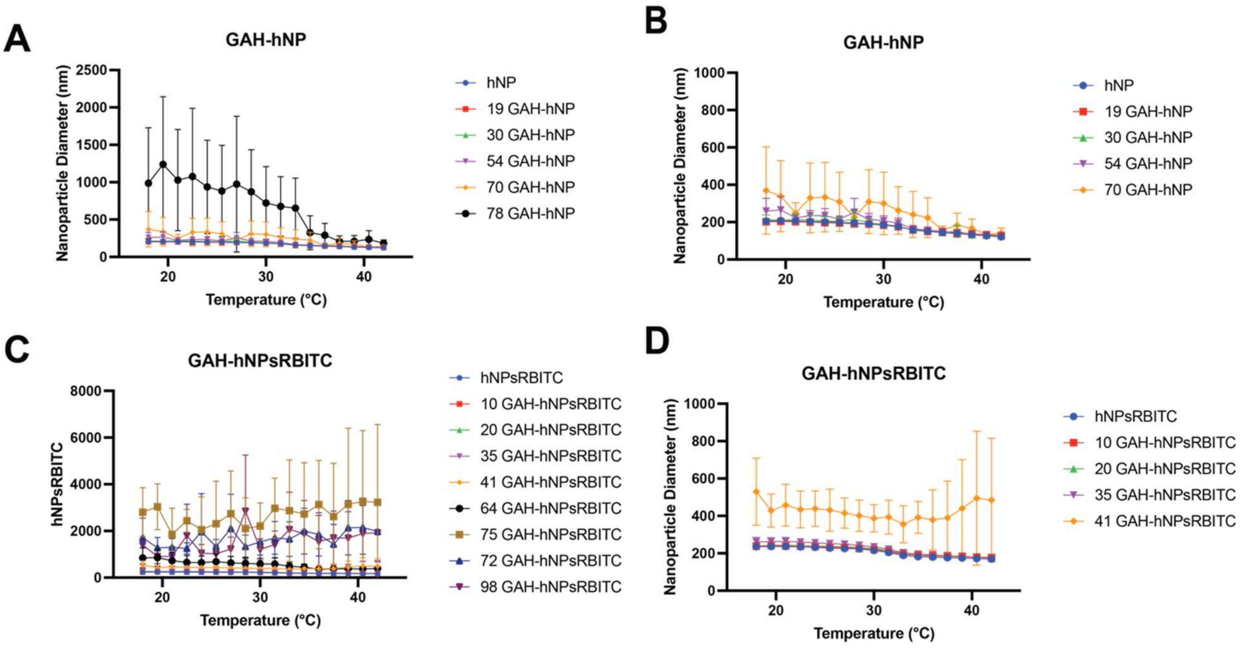

Figure S1. Increasing peptide concentration on the surface of hNP and hNPsRBITC significantly increases the variation in nanoparticle diameter. (**A**) hNP conjugated with 19–78 GAH peptides; (**B**) hNP conjugated with 19–70 GAH peptides; (**C**) hNPsRBITC conjugated with 10–98 GAH peptides; (**D**) hNPsRBITC conjugated with 10–41 peptides

Table S1. GAH-hNP diameter, PDI and Zeta-Potential at increasing GAH concentration on the surface of hNP.

GAH:hNP Conjugation	Diameter 18 °C (nm)	Diameter 42 °C (nm)	Zeta Potential 18 °C (mV)	Zeta Potential 42 °C (mV)	PDI 18 °C	PDI 42 °C
0:1 (hNP)	205.20 ± 8.83	121.57 ± 6.71	-24.93 ± 2.53	-30.22 ± 1.65	0.13 ± 0.03	0.11 ± 0.03
0.5:1 (19 GAH-hNP)	202.46 ± 14.69	128.67 ± 11.34	-9.89 ± 1.66	-12.89 ± 1.87	0.11 ± 0.0	0.09 ± 0.02
1:1 (30 GAH-hNP)	213.43 ± 24.12	125.54 ± 10.03	-9.85 ± 2.82	-14.03 ± 1.08	0.17 ± 0.09	0.14 ± 0.06
2:1 (54 GAH-hNP)	258.99 ± 69.16	126.60 ± 12.27	-13.99 ± 2.04	-17.13 ± 2.15	0.25 ± 0.14	0.18 ± 0.05
4:1 (70 GAH-hNP)	369.84 ± 234.59	140.57 ± 27.98	-11.74 ± 2.77	-13.87 ± 2.40	0.34 ± 0.30	0.23 ± 0.15
6:1 (78 GAH-hNP)	986.36 ± 741.27	182.38 ± 45.18	-8.61 ± 4.03	-11.89 ± 3.39	0.52 ± 0.29	0.30 ± 0.07

Table S2. GAH-hNPsRBITC diameter, PDI, and Zeta-Potential at increasing GAH concentration on the surface of hNPsR-BITC.

GAH: hNPsRBITC Conjugation	Diameter 18 °C (nm)	Diameter 42 °C (nm)	Zeta Potential 18 °C (mV)	Zeta Potential 42 °C (mV)	PDI 18 °C	PDI 42 °C
0:1 (hNPsRBITC)	235.75 ± 3.63	169.60 ± 3.96	-21.41 ± 1.26	-35.11 ± 0.77	0.04 ± 0.02	0.06 ± 0.02
0.5:1 (10 GAH-hNPsRBITC)	238.73 ± 5.33	177.31 ± 5.99	-8.94 ± 1.92	-12.01 ± 2.46	0.07 ± 0.03	0.08 ± 0.02
1:1 (20 GAH-hNPsRBITC)	238.25 ± 8.37	177.52 ± 5.44	-8.69 ± 3.06	-10.44 ± 1.23	0.11 ± 0.08	0.09 ± 0.05
2:1 (35 GAH-hNPsRBITC)	261.41 ± 24.73	175.80 ± 1.69	-7.90 ± 1.43	-10.71 ± 2.57	0.13 ± 0.06	0.12 ± 0.06
4:1 (41 GAH-hNPsRBITC)	529.92 ± 179.26	484.56 ± 330.96	-8.75 ± 1.64	-14.33 ± 0.94	0.49 ± 0.22	0.40 ± 0.15
6:1 (64 GAH-hNPsRBITC)	844.35 ± 424.90	393.14 ± 25.43	-8.45 ± 2.53	-16.33 ± 2.85	0.53 ± 0.15	0.48 ± 0.12
8:1 (75 GAH-hNPsRBITC)	2801.00 ± 1047.58	3223.51 ± 3341.12	-8.18 ± 2.91	-11.57 ± 3.41	0.44 ± 0.08	0.83 ± 0.14
10:1 (72 GAH-hNPsRBITC)	1709.23 ± 1112.62	1975.47 ± 1246.46	-5.44 ± 1.78	-9.31 ± 3.75	0.72 ± 0.26	0.79 ± 0.21
12:1 (98 GAH-hNPsRBITC)	1394.99 ± 1151.76	1926.57 ± 1254.16	-8.31 ± 5.81	-11.37 ± 7.59	0.77 ± 0.22	0.70 ± 0.22

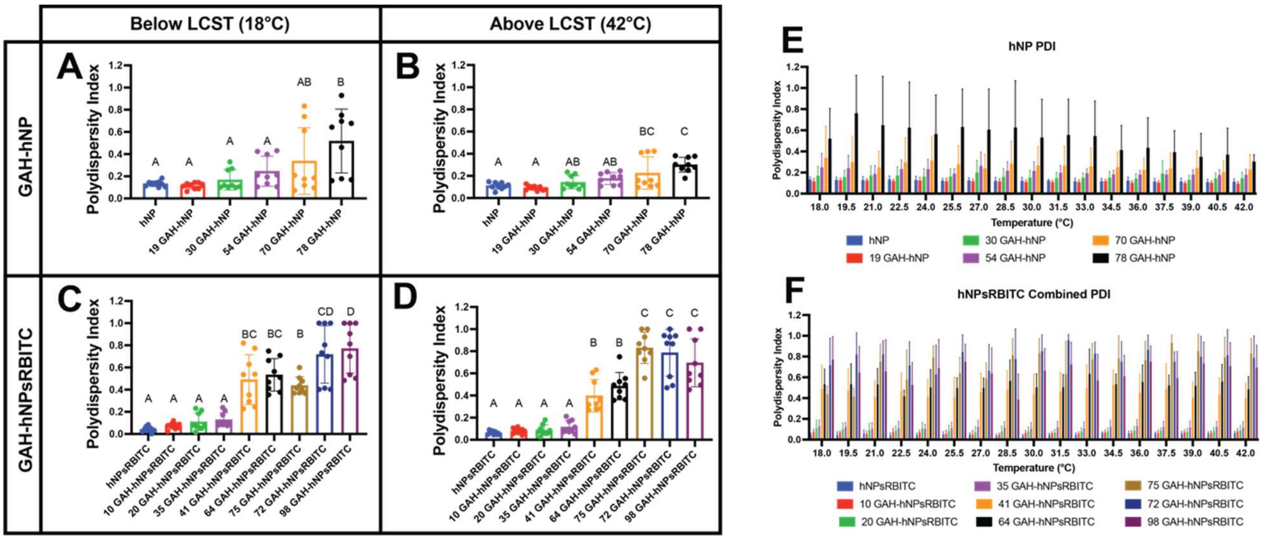

Figure S2 Increasing GAH concentration on the surface of hNP and hNPsRBITC increasing polydispersity of particles in solution. Direct values listed in Supplemental Tables 1 and 2. (**A**) Zeta potential of hNP conjugated with 19–78 peptides at 18 °C; (**B**) zeta potential of hNP conjugated with 19–78 peptides at 42 °C; (**C**) zeta potential of hNPsRBITC conjugated with 10–98 peptides at 18 °C; (**D**) zeta potential of hNPsRBITC conjugated with 10–98 peptides at 42 °C; (**E**) zeta potential of hNP conjugated with 19–78 peptides at 18 °C–42 °C; (**F**) zeta potential of hNPsRBITC conjugated with 10–98 peptides at 18 °C–42 °C;

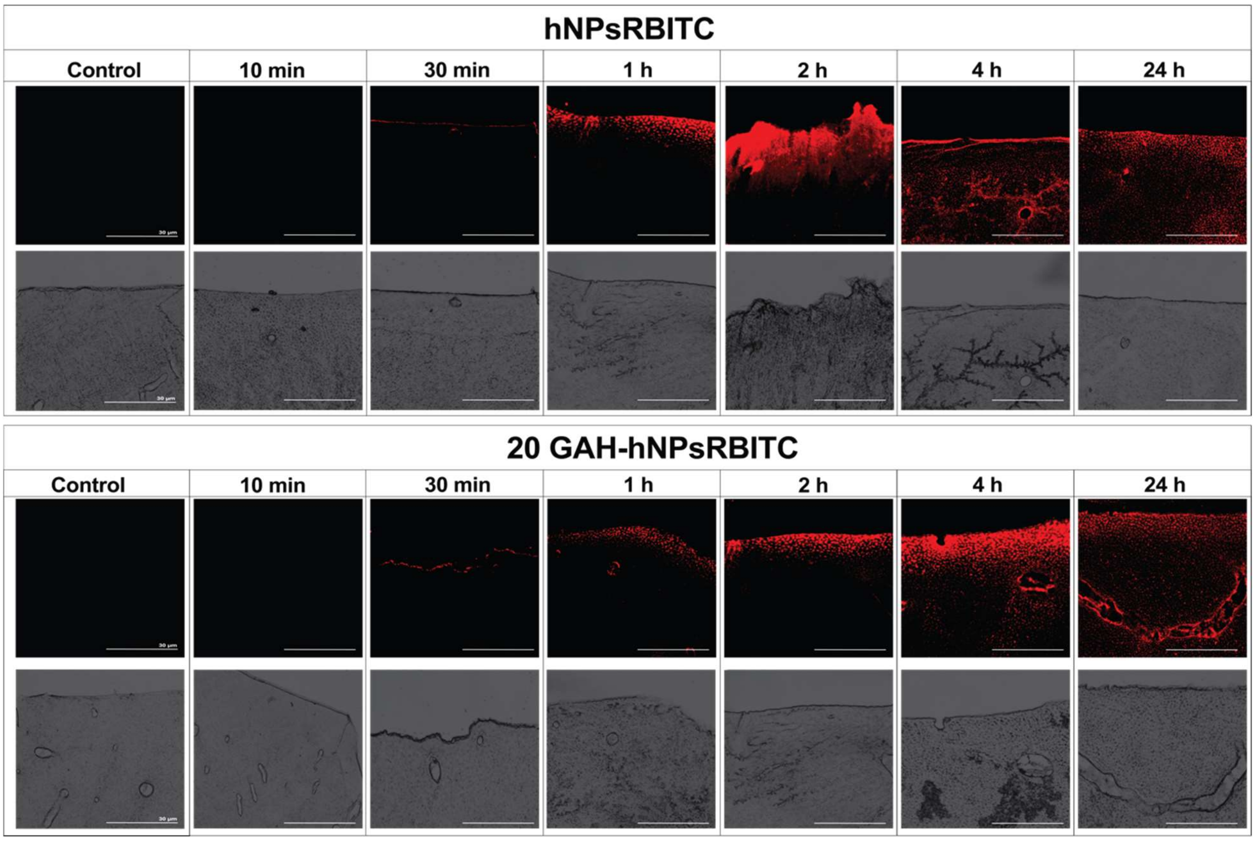

Figure S3. Timed diffusion of 20 GAH-hNPsRBITC into AD cartilage explants.

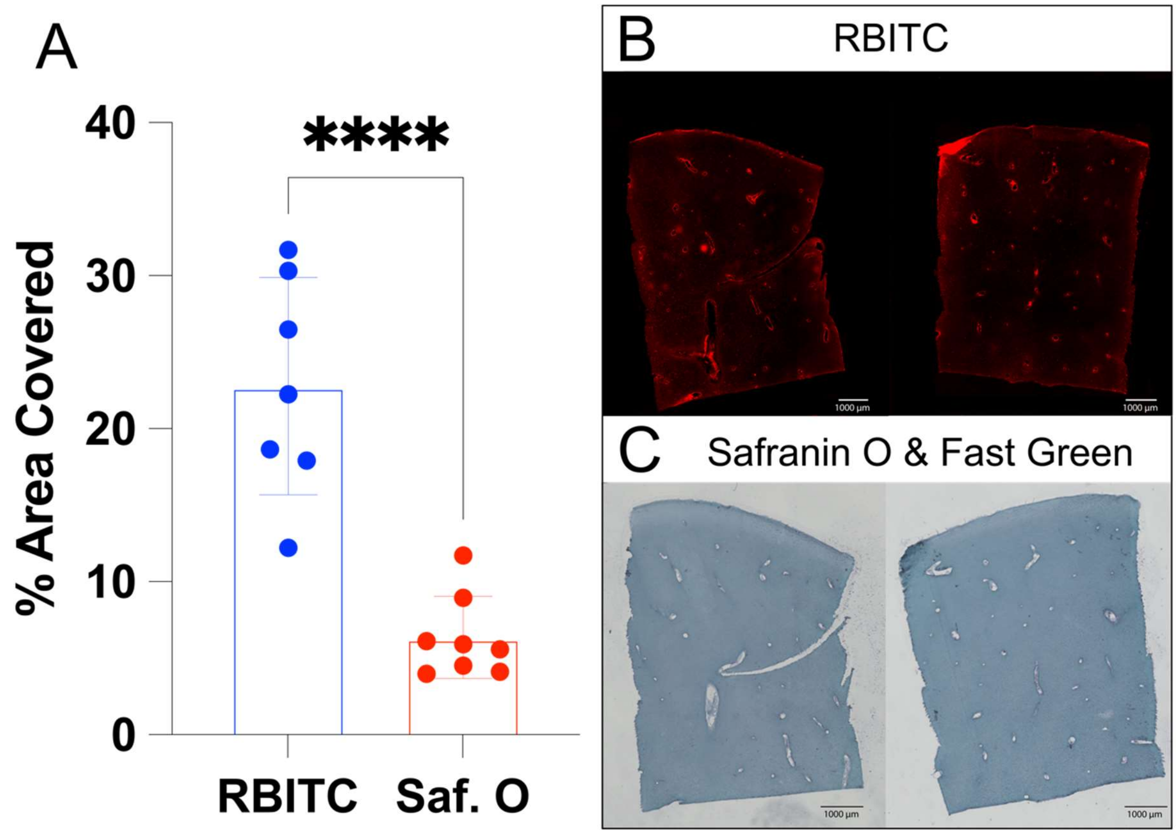

Figure S4. Aggrecan-depleted explants treated with 0.10 mg unconjugated hNPsRBITC, frozen in OCT, and sectioned. The explants were quantified for hNPsRBITC and Safranin O and Fast Green (**A**). Example explants are shown in (**B**) hNPsRBITC and (**C**) Safranin O and Fast Green that were assessed to determine whether the sulfated AMPS within the hNPsRBITC were stained as well. The Safranin O and Fast Green stain does not stain the hNPsRBITC. Scale bars are 1000 µm. $p < 0.0001$ represented by ****.

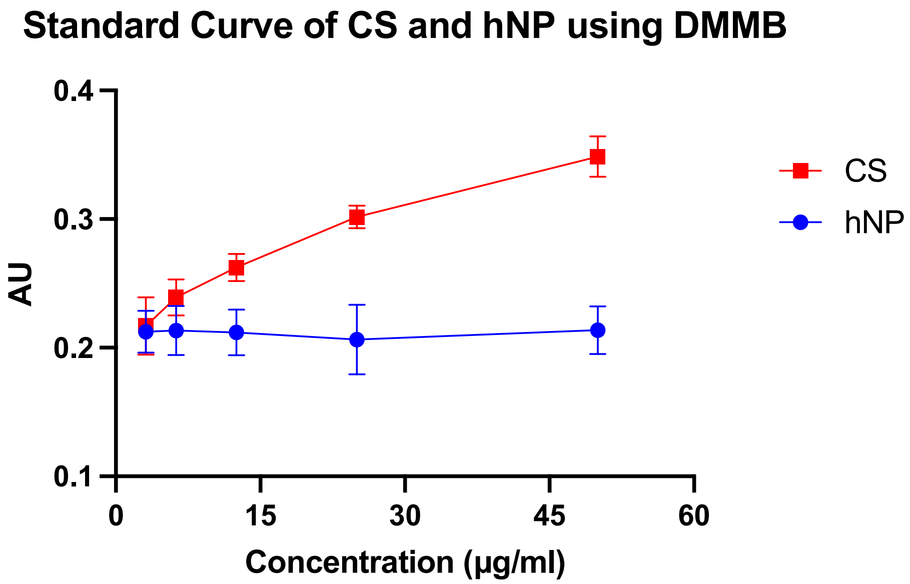

Figure S5. Standard curves of chondroitin sulfate (CS) (red) and hNP (blue) using DMMB assay. CS SC: $y = 0.0028x + 0.2171$ $R^2 = 0.9661$; hNP SC: $0.0002x + 0.2106$ $R^2 = 0.7512$.
